# Biomonitoring of Exposure to Organophosphate Pesticides in New York City

**DOI:** 10.1289/ehp.1408444

**Published:** 2014-07-01

**Authors:** John H. Ross, Michael E. Ginevan

**Affiliations:** 1 risksciences.net, LLC, Carmichael, California, USA; 2 M.E. Ginevan & Associates, Silver Spring, Maryland, USA; E-mail: jhross@surewest.net

In a recent publication, McKelvey et al. (2013) reported higher levels of some urinary metabolites of organophosphate (OP) and pyrethroid insecticides in a cohort from New York City (NYC) compared with a national cohort. However, the authors concluded that

Estimates of exposure to pyrethroids and dimethyl organophosphates were higher in NYC than in the United States overall, underscoring the importance of considering pest and pesticide burdens in cities when formulating pesticide regulations. (McKelvey et al. 2013)

Although urinary excretion levels of dialkylphosphates (DAPs)—which are metabolites and environmental degradates of OP insecticides—are useful as quantitative indicators of exposure to OPs in agricultural workers, they are not as useful for the general population. For agricultural workers, the source of the OP and time of exposure are typically known, and there is substantial exposure above background, thus allowing an unambiguous estimation of exposure levels (Chen et al. 2013). In contrast, urinary excretion levels of DAPs in the general population represent an integrated measurement of intakes of both pesticide and pesticide metabolites 1 or 2 days prior to sampling (Chen et al. 2012). Half-lives of OPs and their metabolites in the body range from a few hours to < 1 day (Chen et al. 2013; Timchalk et al. 2007). Evidence indicates that urinary DAPs in the general population primarily result from exposures to nonneurotoxic OP metabolites that are in food (Krieger et al. 2012).

McKelvey et al. (2013) stated that

Diazinon and chlorpyrifos were detected in all indoor air samples collected from a sample of NYC homes (Whyatt et al. 2007) and from the majority of floor-wipe samples in a national study of U.S. homes (Stout et al. 2009) several years after these compounds were phased out for indoor use.

Measureable human exposure to chlorpyrifos applied indoors declines to background levels within weeks of an application (Krieger et al. 2001), so although measureable residues may remain indoors, those residues do not contribute appreciably to the urinary DAPs from chronic low-level exposure to OP metabolites in food. Likewise, ambient inhalation exposures, even in high-use agricultural areas, contribute negligibly to OP exposure (McKone et al. 2007). The inescapable conclusion is that if OP use in NYC were driving elevated urinary excretion levels, a sizeable percentage of NYC residents would have been exposed to recent indoor uses of dimethyl OPs. Yet, at the time of the sampling, use of these OPs was almost completely phased out. Moreover, although diethyl OPs, such as chlorpyrifos and diazinon, had been used extensively indoors until 2001, the dimethyl OPs—and especially dimethylthio OPs—were never used as extensively indoors, as evidenced by the results reported by Stout et al. (2009).
